# miR-29c overexpression and COL4A1 downregulation in infertile human endometrium reduces endometrial epithelial cell adhesive capacity *in vitro* implying roles in receptivity

**DOI:** 10.1038/s41598-019-45155-6

**Published:** 2019-06-14

**Authors:** Meaghan Griffiths, Michelle Van Sinderen, Katarzyna Rainczuk, Evdokia Dimitriadis

**Affiliations:** 1 Embryo Implantation Laboratory, Hudson Institute of Medical Research, Clayton, Victoria 3168 Australia; 2 Department of Molecular and Translational Medicine, Monash University, Clayton, Victoria 3800 Australia; 3 Department of Anatomy and Developmental Biology, Monash University, Clayton, Victoria 3800 Australia; 4 Department of Obstetrics and Gynaecology, University of Melbourne, The Royal Women’s Hospital, Parkville, Victoria 3010 Australia

**Keywords:** Predictive markers, Translational research

## Abstract

The endometrium is a highly complex tissue that is vulnerable to subtle gene expression changes and is the first point of contact for an implanting blastocyst. Successful blastocyst implantation can only occur when the endometrium is receptive during a short window with each menstrual cycle. microRNAs are small, non-coding RNAs that negatively regulate their gene targets. miR-29c has previously been identified to be differentially regulated across the fertile menstrual cycle, however it has not been investigated in association with infertility. We hypothesised that miR-29c dysregulation in the infertile endometrium would negatively influence endometrial adhesion and blastocyst implantation outcomes during the mid-secretory, receptive phase. miR-29c expression was elevated in early and mid-secretory phase infertile endometrium and localised to the epithelial compartments of endometrial tissue. Overexpression of miR-29c *in vitro* impaired endometrial epithelial adhesion, and reduced collagen type IV alpha 1 (COL4A1) mRNA expression. COL4A1 was immunolocalised to the luminal and glandular epithelial basement membranes in early and mid-secretory phase fertile and infertile endometrium for the first time. Knockdown of COL4A1 impaired endometrial epithelial adhesion suggesting a role in endometrial receptivity and implantation. Our data suggests miR-29c overexpression with infertility may impair the adhesive capacity of the endometrium, potentially contributing to implantation failure and infertility.

## Introduction

Infertility is a global health burden that affects approximately 9% of couples worldwide and is defined by the inability to conceive after 12 months of unprotected sex without the use of contraceptives^1–3^. Although access to *in vitro* fertilisation (IVF) is abundant in developed countries, success rates have stagnated to around 30%^4^. There are also considerable financial costs to consider with Australian couples spending on average ~$35,000 on multiple IVF cycles before they achieve a live birth^5^. Therefore, it is important to investigate how IVF success rates can be improved to ease this burden on world health care and help infertile couples achieve a successful pregnancy.

For successful pregnancy to be achieved, blastocyst implantation must occur and is initiated via the firm adhesion of a blastocyst to an adequately receptive endometrial epithelium. While blastocyst quality has been extensively studied, equally as important is the preparation of the uterine lining, the endometrium.

Endometrial receptivity refers to the finite window in each menstrual cycle in which the endometrium is sufficiently prepared for an implanting blastocyst^6^. If the endometrium is not receptive, blastocyst implantation fails. The receptive phase coincides with the mid-secretory phase of the menstrual cycle, approximately 7–10 following ovulation, or days 19–23 of the average 28-day menstrual cycle. The endometrium is a highly complex tissue that is extensively remodelled throughout each menstrual cycle, and therefore it is likely vulnerable to changes that occur early in the cycle that may impair endometrial remodelling and the attainment of receptivity and embryo implantation.

There are many known regulators of human receptivity, including leukemia inhibitory factor (LIF) and interleukin 11 (IL-11)^7–10^, and more recently microRNA (miR) have been implicated to regulate endometrial receptivity and blastocyst implantation^11^. miR have been identified to be expressed and secreted by both human endometrium and blastocysts and consequently taken up by the opposite to have downstream effects on gene targets^12–14^.

miR are short, 20–24 nucleotide, non-protein coding lengths of RNA involved in post-transcriptional gene regulation^15^. miR act by binding to the 3′ untranslated region (UTR) of target genes to induce degradation of mRNA transcripts or translational repression of the protein product^16^.

The miR-29 family consists of miR-29a, b, and c, distributed across two chromosomes, 1q32 and 7q32^17^. miR-29c is well established as a tumour suppressor in many cancers including lung^18^, gastric^19^, pancreatic^20^, and bladder^21^. Within the endometrium, miR-29c is upregulated in the mid-secretory, receptive phase compared to proliferative phase endometrium of fertile women^22^, and is significantly reduced in the ectopic endometrium compared to eutopic endometrium of women with endometriosis^23^.

miR-29c has not be investigated in relation to female infertility and endometrial receptivity. The rationale of this study was to define the localisation and determine the expression levels of miR-29c in the early secretory, pre-receptive phase endometrium from fertile and infertile women. We hypothesised that miR-29c is dysregulated in the infertile endometrial epithelium and subsequently alters its target gene expression within the epithelium, altering the attainment of receptivity, endometrial epithelial adhesive capacity and implantation outcomes.

Due to the difficulty in studying human endometrial receptivity *in vivo*, endometrial epithelial cells cultured *in vitro* from primary endometrial biopsies are a commonly utilised model^13^. Moreover, due to species differences, mouse models of receptivity are also limited in their usefulness due to differences in gene expression profiles required for mouse receptivity^24^. For these reasons, we use primary human endometrial epithelial cells cultured *in vitro* as a model of endometrial receptivity here.

## Results

### miR-29c expression is elevated in early and mid-secretory phase infertile human endometrium, compared to fertile controls, and localises to the endometrial epithelium

**Figure 1:**
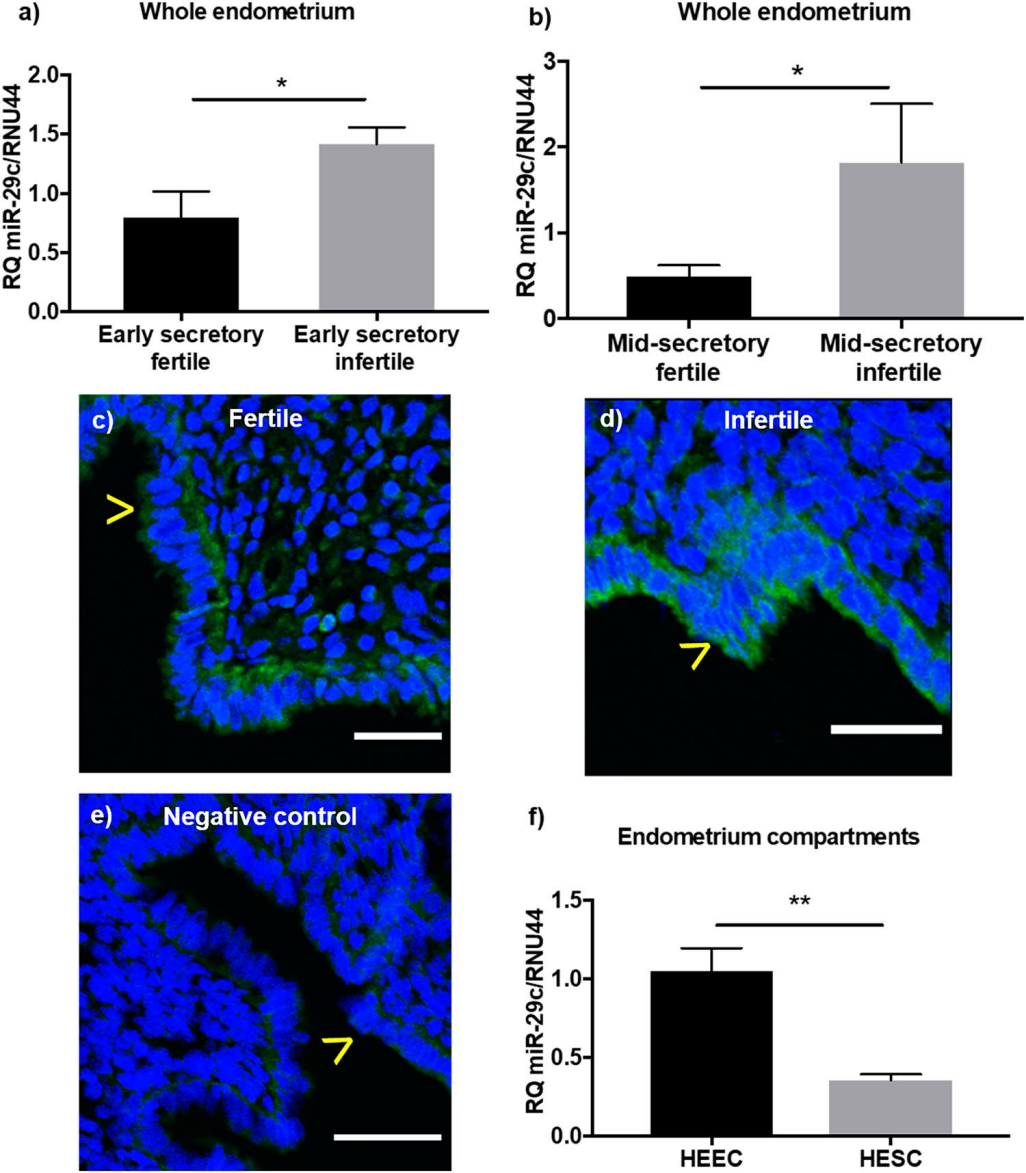
miR-29c expression in the endometrium. miR-29c is significantly overexpressed in infertile endometrium from the (**a**) early (p = 0.03) and (**b**) mid-secretory (p = 0.01) phases compared to fertile endometrium of the same phase (n = 6–10/group). (**c**) miR-29c localises to the luminal and glandular epithelial compartments of the endometrium, depicted by green fluorescent staining, in both (**c**) fertile and (**d**) infertile women, compared to negative control (**e**). (n = 5/group) (**f**) miR-29c expression is significantly (p = 0.009) greater in isolated endometrial epithelial cells compared to endometrial stromal cells (n = 3/group). Data presented as mean ± SEM. Mann-Whitney test (**a**,**b**) and unpaired t-test (**f**) for significance (*p < 0.05, **p < 0.01). Scale bars represent 50 µm. (>luminal epithelium).

Relative quantification with qPCR revealed miR-29c expression levels were elevated in early (Fig. 1a) and mid-secretory (Fig. 1b) phase infertile whole endometrial tissue compared to fertile endometrium of the same phase (Fig. 1a; p = 0.03; n = 8–10/group; fertile 1.08 ± 0.37, infertile 3.20 ± 0.94 and Fig. 1b; p = 0.01; n = 6/group; fertile 0.49 ± 0.13, infertile 1.82 ± 0.69).

miR-29c localisation in the endometrium was determined using *in situ* hybridisation which revealed localisation to the luminal epithelium of fertile (Fig. 1c) and infertile (Fig. 1d) endometrium. Minimal localisation in the endometrial stromal compartment in both fertile and infertile endometrium was found (Fig. 1c,d). Investigation of miR-29c expression in isolated primary human endometrial epithelial (HEEC) and stromal (HESC) cells revealed significantly higher miR-29c expression in HEEC compared to HESC (Fig. 1f; p = 0.009; n = 3/group; HEEC 1.05 ± 0.14, HESC 0.35 ± 0.04).

### miR-29c overexpression in primary endometrial epithelial cell monolayers impaired HTR8/SVneo trophoblast cell line spheroid adhesion

To determine the functional role of miR-29c overexpression in the context of endometrial receptivity and blastocyst implantation, HEEC monolayers were transiently transfected with synthetic miR-29c mimic. A HTR8/SVneo spheroid adhesion assay was performed 72 hours later to emulate blastocyst implantation in the mid-secretory, receptive phase.

**Figure 2:**
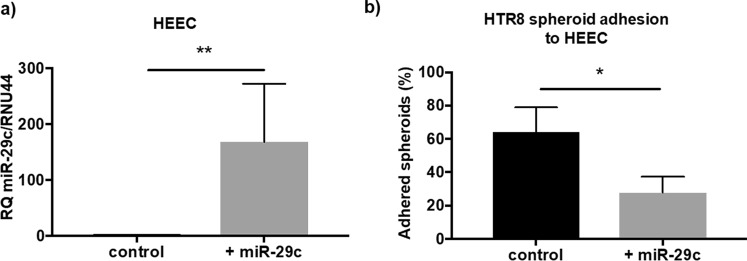
Function of miR-29c overexpression. (**a**) Confirmation of miR-29c overexpression using a synthetic miR mimic (+miR-29c) in primary human endometrial epithelial cells (HEEC), compared to control (p = 0.008; n = 8/group; Wilcoxon signed rank test). (**b**) miR-29c overexpression (+miR-29c) in a HEEC monolayer significantly (p = 0.04) reduces HTR8/SVneo trophoblast spheroid adhesion compared to control (n = 5; paired t-test). Data are presented as mean ± SEM. Wilcoxon signed-rank test (**a**) paired t-test performed (**b**) for significance (*p < 0.05, **p < 0.01).

miR-29c overexpression in isolated HEEC was confirmed by qPCR shown in Fig. 2a (p = 0.008; n = 8; + miR-29c mimic 168.1 ± 104.2, scrambled control 1.05 ± 0.23). miR-29c overexpression in a HEEC monolayer significantly reduced HTR8/SVneo trophoblast spheroid adhesion after two hours, compared to control (Fig. 2b; p = 0.04; n = 5; control 64.28% ± 14.61, + miR-29c 27.78% ± 9.54).

### miR-29c overexpression negatively regulates predicted target gene COL4A1

miRs negatively regulate their gene targets by binding to the 3′UTR to induce mRNA degradation or translational repression^16^. Confirmed and predicted miR-29c targets were identified using bioinformatics programs DIANA, TarBase, and TargetScan as previously described^13^.

**Figure 3:**
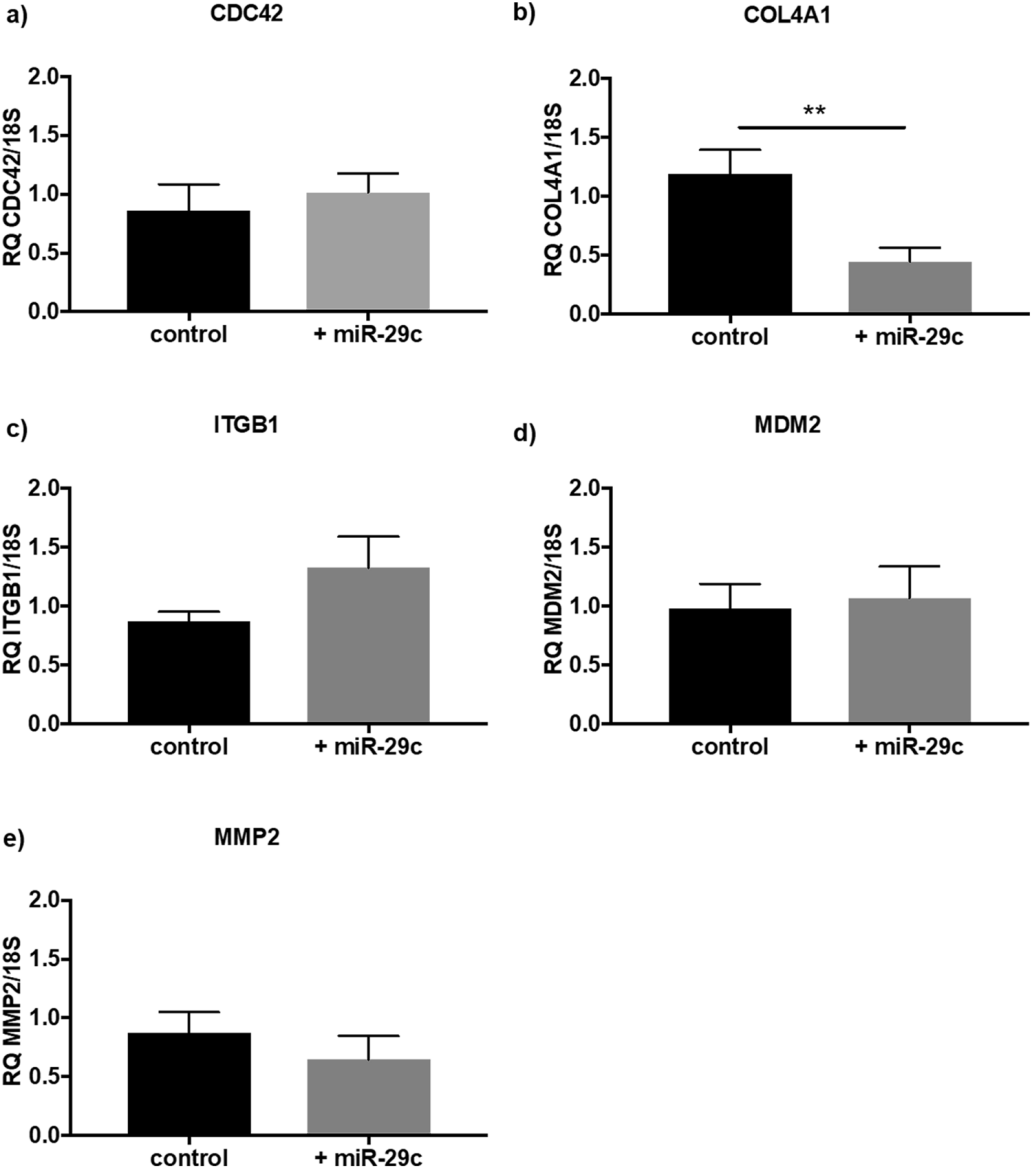
Regulation of miR-29c target gene expression. miR-29c overexpression ( +miR-29c) in HEEC (n = 6/group) elicits (**a**) no change in CDC42, (**b**) significant (p = 0.002) reduction in COL4A1 mRNA, (**c**) no change in ITGB1, (**d**) no change in MDM2, and (**e**) no change in MMP2. Data are presented as mean ± SEM. Paired t-tests for significance (**p < 0.01).

The expression of the miR-29c targets CDC42, COL4A1, ITGB1, MDM2, and MMP2 were determined using qPCR and are shown in Fig. 3. When miR-29c was overexpressed in HEEC, COL4A1 mRNA expression was significantly reduced compared to control (Fig. 3b; p = 0.002; n = 6; control 1.19 ± 0.2, +miR-29c 0.45 ± 0.11). No change was observed in COL4A1 mRNA expression in whole endometrial tissue (see Supplementary Fig. S1). No changes in gene expression levels were observed for all other miR-29c target genes in response to miR-29c overexpression (CDC42 p = 0.56, ITGB1 p = 0.19, MDM2 p = 0.77, MMP2 p = 0.28).

### COL4A1 protein localises to the luminal epithelium basement membrane and is reduced in early and mid-secretory infertile endometrium

**Figure 4:**
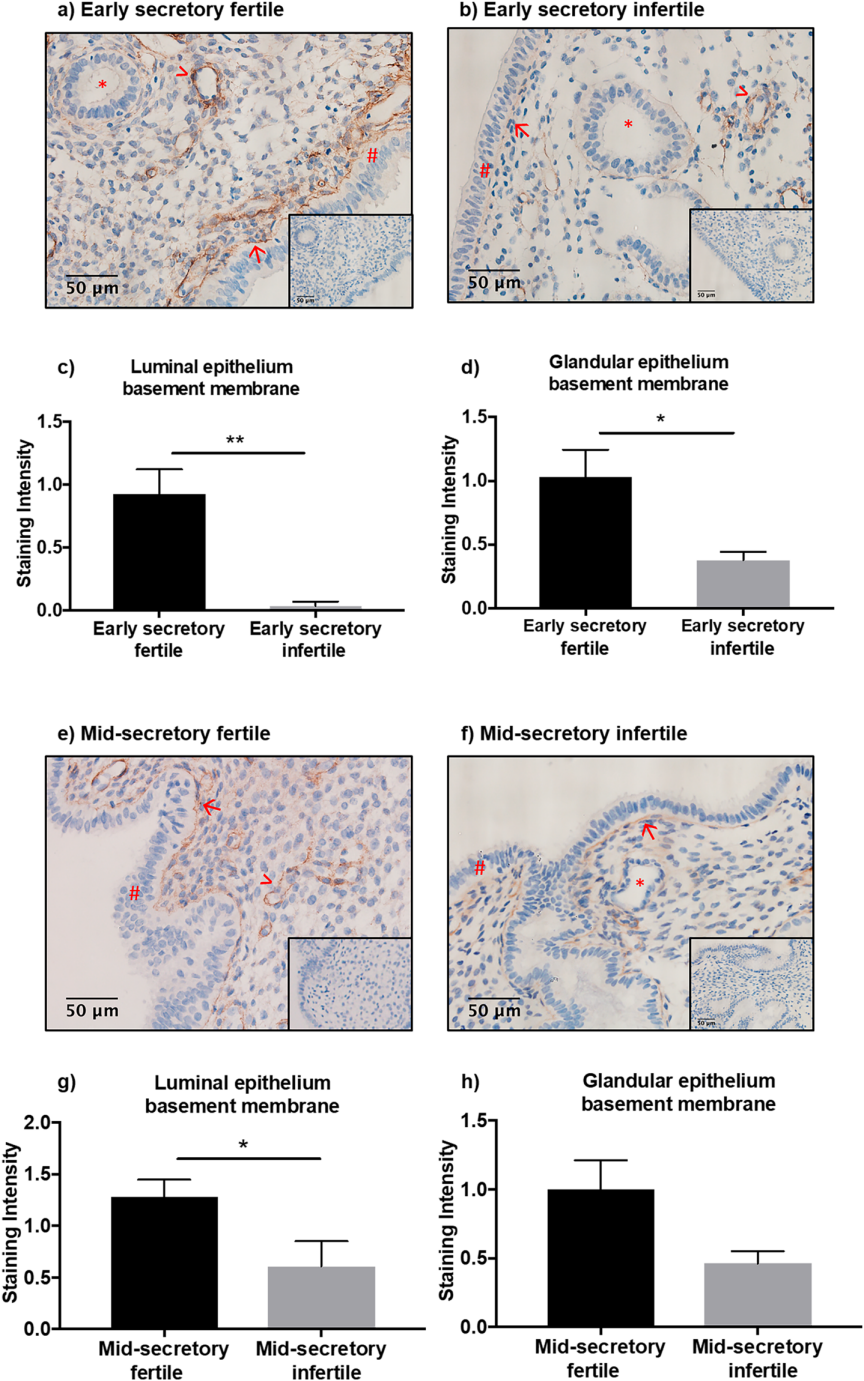
Collagen type IV alpha 1 (COL4A1) protein localisation in the endometrium. COL4A1 localises to the luminal and glandular epithelium basement membranes in early secretory (**a**,**b**) and mid-secretory (**e**,**f**) fertile and infertile endometrium (>endothelial cells, → luminal epithelium basement membrane, #luminal epithelium, *glandular epithelium, S stroma). Staining intensity was semi-quantitated and showed (**c**) significantly lower COL4A1 protein localisation to the luminal epithelium basement membrane (p = 0.005), and (**d**) glandular epithelium basement membrane (p = 0.04) in early secretory phase infertile endometrium, compared to fertile. COL4A1 localisation is also lower in luminal epithelium basement membrane of mid-secretory phase infertile endometrium (**g**), but not glandular epithelium basement membrane (**h**). Data are presented as mean ± SEM. Mann Whitney tests (**a**,**b**,**h**) and unpaired t-test (**g**) for significance.

To determine COL4A1 localisation and relative protein levels in the endometrium of fertile and infertile women, immunohistochemistry was performed. COL4A1 protein localised to the luminal and glandular epithelium basement membranes (Fig. 4) in early and mid-secretory phase endometrium from fertile and infertile women. The COL4A1 immunoprotein staining intensity was semi-quantitated by scoring COL4A1 staining in tissues blinded to the cycle stage and fertility status. The luminal epithelium basement membrane staining was significantly lower in early secretory infertile endometrium, compared to early secretory fertile endometrium (Fig. 4c; p = 0.005; n = 7/group; fertile 0.93 ± 0.19, infertile 0.04 ± 0.04). Glandular epithelial basement membrane localisation was also significantly lower in early secretory infertile, compared to fertile endometrium (Fig. 4d; p = 0.046; n = 8/group; fertile 1.03 ± 0.21, infertile 0.38 ± 0.07). In mid-secretory phase endometrium, the luminal epithelium basement membrane staining was significantly reduced in the infertile endometrium (Fig. 4g; p = 0.04; n = 7–8/group; fertile 1.28 ± 0.17, infertile 0.61 ± 0.24). No difference was observed in mid-secretory glandular epithelium basement membrane COL4A1 localisation (Fig. 4h; p = 0.07; n = 7–8/group; fertile 1.0 ± 0.60, infertile 0.46 ± 0.09).

Semi-quantitation of COL4A1 immunostaining intensity in the stromal and endothelial compartments was also completed (see Supplementary Fig. 2), and no differences were seen between fertile and infertile endometrium respectively.

### COL4A1 mRNA knockdown in a HEEC monolayer significantly alters HTR8/SVneo trophoblast spheroid adhesion

Once we established that miR-29c negatively regulates COL4A1 mRNA in the early secretory infertile endometrium, we aimed to see if knockdown of COL4A1 *in vitro* would reduce HEEC adhesion, similarly to miR-29c overexpression.

**Figure 5:**
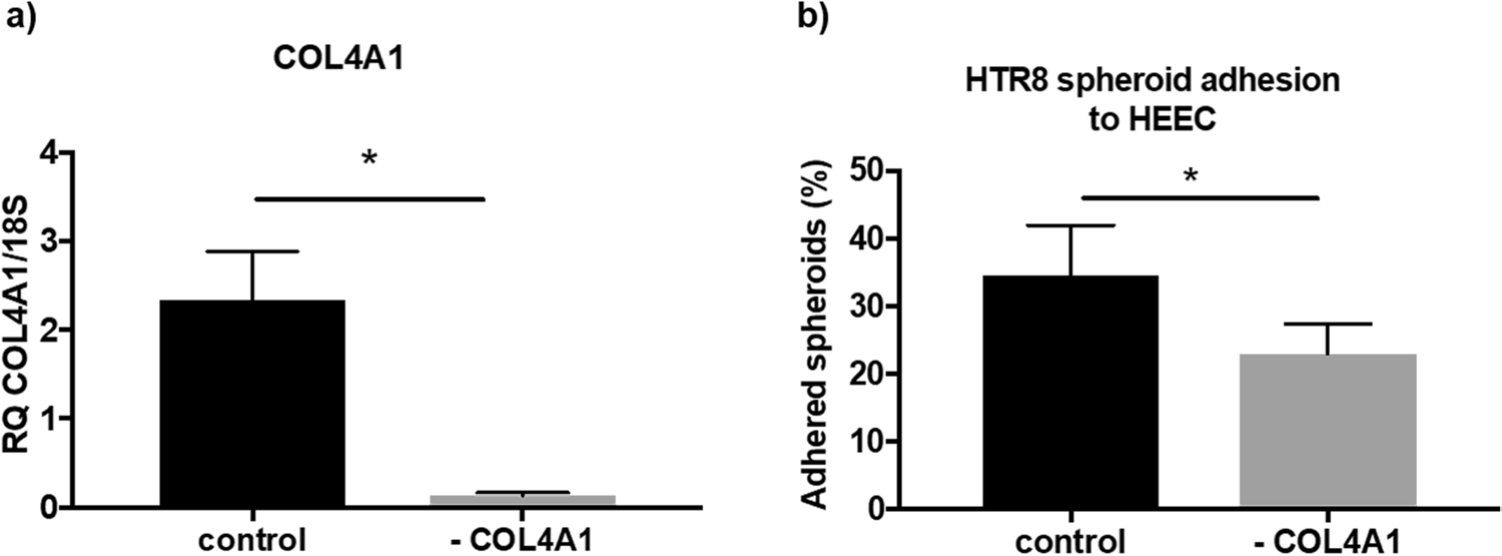
Function of COL4A1 knockdown. (**a**) COL4A1 siRNA transfection significantly (p = 0.02) reduces COL4A1 mRNA expression in HEEC (n = 5). (**b**) Knockdown of COL4A1 in HEEC monolayer significantly (p = 0.03) impairs HTR8/SVneo spheroid adhesion (n = 6). Data are presented as mean ± SEM. Paired t-tests (**a**) and Wilcoxon signed rank test (**b**) for significance.

siRNA knockdown of COL4A1 significantly reduced COL4A1 levels in HEEC (Fig. 5a; p = 0.02; n = 6; control 2.34 ± 0.55, COL4A1 knockdown 0.13 ± 0.03). COL4A1 siRNA knockdown did not alter mRNA expression of COL4A2, COL4A3, COL4A4, COL4A5, COL4A6 in HEEC (see Supplementary Fig. 3).

To determine if COL4A1 siRNA knockdown reduces endometrial epithelial adhesion, independent of miR-29c overexpression HTR8/SVneo trophoblast spheroid adhesion assays were completed. COL4A1 knock down in a HEEC monolayers significantly reduced HTR8/SVneo spheroid adhesion (Fig. 5b; p = 0.03; n = 6; control 34.48 ± 7.52, COL4A1 knockdown 22.95 ± 4.51).

## Discussion

This is the first study to investigate the expression and function of miR-29c in the endometrium in relation to infertility. We show for the first time, miR-29c expression is significantly elevated in the early secretory and mid-secretory phase endometrium of infertile women compared to fertile endometrium of the same phase. Furthermore, we show miR-29c localises to the luminal epithelium of fertile and infertile endometrium. We demonstrate miR-29c overexpression *in vitro* significantly impairs the adhesive capacity of HEEC and we propose this may occur via the significant downregulation of COL4A1, which also results in impaired endometrial epithelial adhesive capacity when reduced in HEEC *in vitro*.

Previously, miR-29c has only been identified to be differentially regulated across the fertile menstrual cycle with two array studies showing miR-29c expression in fertile endometrial epithelial cells and endometrial fluid is elevated in the mid-secretory phase compared to the proliferative phase^12,22^. This is the first study to show miR-29c dysregulation in human endometrium with infertility.

Our *in vitro* functional studies demonstrate miR-29c overexpression impairs trophoblast spheroid adhesion, suggesting it may act during the secretory phase of infertile women to impair receptivity and implantation success. We hypothesised miR-29c overexpression observed in the infertile endometrium yields functional effects during the receptive phase to impair implantation, and to support this we demonstrate a functional deficit on trophoblast spheroid adhesion 72 hours after transfection with miR-29c mimic, suggesting miR-29c overexpression impairs endometrial cell adhesion, which is a critical process for successful implantation.

miR exert functional changes by post-transcriptionally regulating their gene targets by mRNA transcript degradation or translational repression. Here, we used bioinformatics programs to identify miR-29c targets of interest and searched the literature for previously published confirmed miR-29c targets.

We assessed five bioinformatically predicted and experimentally confirmed miR-29c targets. miR-29c has been investigated in a number of cancer settings with a number of confirmed targets identified, including integrin beta 1 (ITGB1) and matrix metalloproteinase 2 (MMP2) for which luciferase reporter assays confirming direct targeting of miR-29c has been previously reported^18,19,25^. Predicted miR-29c targets we assessed here include CDC42, COL4A1, and MDM2. In the current study, we screened for potential miR-29c targets by observing changes in mRNA transcript levels. This approach has limitations, namely it only allows for identifying targets that undergo mRNA degradation in response to miR-29c regulation, neglecting targets that are regulated by translational repression.

Cell division cycle 42 (CDC42) has not been previously published in association with miR-29c however, silencing CDC42 in a HESC *in vitro* model of implantation has been shown to reduce mouse blastocyst implantation to HESC^26^. Here, we saw no difference in CDC42 mRNA expression levels in HEEC where miR-29c was overexpressed. This may suggest miR-29c does not target CDC42 in the human endometrial epithelium or, CDC42 may be involved in the HESC invasion during blastocyst implantation, rather than the initial adhesive interaction between blastocyst and HEEC of interest in the current study.

Expression of beta integrins fluctuates across the menstrual cycle, increasing during implantation^27^. ITGB1 has previously been confirmed by luciferase assay to be targeted by miR-29c in pancreatic, lung, and gastric cancers^18,19,25^. We saw no difference in ITGB1 expression in response to miR-29c overexpression in HEEC.

Mouse double minute homolog 2 (MDM2) has not been previously investigated in association with miR-29c, however, MDM2 protein localises to the luminal epithelium of cycling endometrium and MDM2 knock down impairs HEEC adhesive capacity^14^. However, in the current study MDM2 mRNA remains unchanged when miR-29c is overexpressed in HEEC, suggesting it may not be targeted by miR-29c for mRNA degradation.

MMP2 is a confirmed miR-29c target in lung cancer^18^. Its expression is reported to be highest in the fertile proliferative phase, suggesting a role in endometrial remodelling^28^. It has also been reported MMP2 expression during receptivity is dysregulated with infertility^29^. However, we saw no difference in MMP2 expression when miR-29c is overexpressed, suggesting MMP2 may not be targeted by miR-29c for mRNA degradation in the cycling endometrium.

Collagen type IV alpha chain 1 (COL4A1) mRNA has been shown to be suppressed by miR-29c overexpression in endothelial cells in an abdominal aortic aneurysm study^30^. COL4A1 is a predicted target of miR-29c. Elevated levels of COL4A1 have also been associated with severe cases of pre-eclampsia^31^. In the present study, COL4A1 mRNA was significantly reduced in HEEC where miR-29c is overexpressed, suggesting miR-29c may negatively regulate COL4A1 *in vivo*.

COL4A1 is encoded in human chromosome 13 alongside COL4A2 which together form a triple helix isoform that is present in all basement membranes^32^. Collagen type IV forms critical basement membrane scaffolding that allows other proteins to be incorporated in the basement membrane and contribute to cell adhesion to the basement membrane via interactions with integrin receptors^32,33^. Collagen type IV knock out studies in mice have shown embryonic lethality occurs relatively late, at E10.5-11.5, attributed by the authors to impaired basement membrane integrity, rather than impaired basement membrane formation^34^. Importantly, changes in the production of COL4A1 in the cellular basement membrane can alter the cell’s adhesive capacity and make it more permissive to adhesion and invasion^33^.

In the context of female reproduction, it is quite well established that COL4A1 is upregulated in the placenta during first trimester pregnancy^35^, and has been shown to positively correlate with preeclampsia severity^31^. A follow-up study by the same authors correlated plasma levels of the COL4A1 fragment, arresten with pre-eclampsia severity providing a potential diagnostic biomarker for preeclampsia^36^.

In normal secretory phase fertile endometrium, COL4 (encompassing all six alpha chains) has been localised to the glandular epithelium basement membrane and endothelial cells of the blood vessels^37^, however, to the best of our knowledge, COL4A1 has not been localised to the infertile endometrium. In the present study, we identified COL4A1 mRNA is reduced in HEEC where miR-29c is overexpressed, suggesting miR-29c acts post-transcriptionally to induce mRNA degradation of COL4A1. To determine if increased miR-29c *in vivo* may correlate with COL4A1 protein levels we immunolocalised COL4A1 in the early and mid-secretory phase infertile endometrium, which had not previously been reported.

COL4A1 localises to the luminal and glandular epithelium basement membranes of early and mid-secretory phase fertile and infertile endometrium. Immunostaining intensity revealed a significant reduction in COL4A1 staining intensity in the luminal epithelium basement membrane in the early and mid-secretory phase infertile endometrium compared to fertile endometrium. Similarly, lower staining intensity was found in the glandular epithelial basement membrane in the early secretory phase infertile endometrium, compared to fertile. These findings are consistent with miR-29c overexpression evident in the early and mid-secretory phase infertile endometrium, and suggest that miR-29c overexpression may translationally repress COL4A1 protein in the early and mid-secretory phase endometrium. Whether miR-29c directly regulates or indirectly alters the expression of COL4A1 remain to be demonstrated, although our data indicates an inverse correlation between miR-29c and COL4A1 levels in endometrial tissue and in primary HEEC. Furthermore, COL4A1 is a predicted target of miR-29c also suggesting that in the endometrium, miR-29c may directly target COL4A1, though we only report an association in the present study.

To understand whether COL4A1 mRNA levels alter endometrial function, we used COL4A1 siRNA to knockdown endogenous COL4A1 levels in HEEC and performed HTR8/SVneo spheroid adhesion assays. COL4A1 siRNA knockdown in a HEEC monolayer substantially reduced HTR8/SVneo spheroid adhesion, demonstrating both miR-29c overexpression and COL4A1 knockdown result in the same functional effect of impaired endometrial adhesion, which suggests miR-29c alters HEEC adhesive capacity in part via downregulation of COL4A1.

This is the first evidence miR-29c is abnormally upregulated in the early and mid-secretory phase infertile endometrium. As miRs are capable of targeting up to thousands of different mRNAs *in vivo*, this finding alone is significant in furthering our understanding of dysregulated genes contributing to infertility. Furthermore, we demonstrate an association with reduced COL4A1 mRNA *in vitro* following miR-29c overexpression in primary HEEC. Functionally, overexpression of miR-29c or downregulation of COL4A1 in HEEC *in vitro* decreases HEEC adhesive capacity. Our data demonstrate that alterations in miR-29c or COL4A1 levels alter primary HEEC adhesive capacity and suggest that the interactions between miR-29c and COL4A1 may impair HEEC adhesive capacity by disrupting basement membrane integrity, contributing to implantation failure and infertility. Targeting COL4A1 or miR-29c *in vivo* may be useful in facilitating implantation in women and furthering our understanding of human infertility, however further studies are required to establish an *in vivo* role.

## Methods

### Primary tissue collection and endometrial epithelial cell isolation

Endometrial tissue was collected at Monash Medical Centre (Clayton, Victoria) from women in the early and mid-secretory phases of their menstrual cycle. Written informed consent was obtained from each patient, with protocols approved by and in accordance with the Monash Health Human Research Ethics Committee, Melbourne, Australia. Fertile women (n = 6-10/phase) had proven parity, while infertile women (n = 7/phase) had primary unexplained infertility. The women had received no steroid treatment for at least two months prior to tissue collection. The collected tissues were examined by an experienced gynaecological pathologist to confirm cycle stage and absence of endometrial dysfunction.

Primary human endometrial epithelial cells (HEEC) were isolated from whole endometrial tissue as previously published^13,14^ and cultured in DMEM/F12 (Thermo Electron Corporation, Melbourne, Australia) medium with 10% fetal calf serum (FCS; Invitrogen, Carlsbad, CA, USA) and 1% antibiotic-myotic solution (Gibco, Auckland, NZ).

### miR-29c mimic and COL4A1 siRNA transfection

HEEC were cultured in 48-well or 96-well plates to ~70% confluence and transfected according to the manufacturer’s instructions using Lipofectamine RNAiMAX and miR-29c mimic (10 nM; Life Technologies) or COL4A1 siRNA (10 nM; Dharmacon) for 72 h. Scrambled sequences (Life Technologies and Dharmacon) were used as negative controls.

### HTR8/SVneo spheroid adhesion assay

Spheroids were made using the HTR8/SVneo cell line (2000 cells/spheroid)^13,14^ in a Cellstar U-shaped 96-well suspension culture plate and incubated for 48 h prior to use in assay. Spheroids were harvested and transferred to transfected HEEC monolayer (10 spheroids/well of 96-well plate, 20 spheroids/well of 48-well plate). Spheroid number per well was counted and recorded using a light microscope before incubation at 37 °C for 2 hours. Following incubation, wells were gently washed with serum-free DMEM/F12 to remove non-adherent spheroids. The remaining spheroids were counted, and a percentage of adhered spheroids calculated^14^.

### RNA isolation and real time quantitative PCR

RNA was isolated from HEEC or whole endometrial tissue biopsies using TRIReagent (Sigma) following the manufacturer’s instructions. RNA concentrations were determined using NanoDrop spectrophotometry. RNA was then reverse transcribed using the TaqMan^TM^ microRNA primer sets (Applied Biosystems) or Superscript III First strand synthesis kit (Invitrogen) for non-microRNA genes to yield complimentary DNA. Real-time qPCR was then performed using the TaqMan^TM^ Universal Master mix II no UNG (Applied Biosystems) or SYBR Power (Applied Biosystems) in combination with TaqMan^TM^ miR primer sets or specific Oligo primer pairs (Sigma). Expression levels were normalised to RNU44 and 18 s respectively, for microRNA and target gene analysis. Relative expression levels were then calculated using comparative cycle threshold method (ΔΔCt).

### *In situ* hybridisation

All *in situ* hybridisation buffers were prepared in diethyl pyrocarbonate (DEPC) treated H_2_O and autoclaved prior to use to remove all RNase activity. Paraffin sections were cut in RNase free environment to prevent endogenous degradation of microRNA.

Protocol was performed following the miRCURY LNA miRNA ISH Optimisation kit Handbook (Qiagen) for formalin fixed, paraffin embedded tissues, with the following details optimised for the miR-29c probe. Proteinase K incubation was performed at 37 °C for 10 minutes. miR-29c custom detection probe (100 nM), LNA U6 snRNA positive control probe (80 nM), and LNA scrambled negative control probe (100 nM) hybridisation was performed for 60 minutes at 60 °C. Blocking was performed for 15 minutes at room temperature with 10% CAS block (Invitrogen) 2% sheep serum and 1% bovine serum albumin (BSA) in PBS with 0.1% Tween. Fluorescent anti-DIG reagent (Sigma-Aldrich) was diluted 1:50 in a diluent solution containing PBS and 1% sheep serum and BSA, and incubated on sections for 60 minutes at room temperature. Nuclear stain was performed with DAPI (ThermoScientific) diluted 1:1000 for 5 minutes. Sections were mounted using fluorescence mounting medium (Dako). After 24 hours, sections were imaged using confocal microscopy. Scale bars were added in Fiji Image software.

### Immunohistochemistry

Sections were dewaxed in Xylene (Sigma) and rehydrated in graded ethanol. Antigen retrieval was performed with heated citrate buffer (pH 6.0) prior to an endogenous peroxidase block using 3% hydrogen peroxide in methanol. Following tris buffered saline (TBS) washes, non-immune blocking was completed with 10% horse serum (Sigma) and 2% human serum in TBS for 1 hour at room temperature, under agitation. COL4A1 antibody (LSBio LS-C312418) was added at 2.0 µg/mL overnight at 4 °C, with a concentration matched mouse IgG (Dako) for each. Sections were thoroughly washed with TBS Tween 0.6% 2 times and once with TBS before sections were incubated in biotinylated horse anti-mouse secondary antibody (Vector) for 30 minutes at room temperature. Simultaneously, avidin-biotin complex (ABC; Vector) was prepared and incubated in the dark for at least 30 minutes prior to application. ABC was added for 30 minutes at room temperature following extensive TBS Tween 0.6% and TBS washes. Diaminobenzidine (DAB; Dako) was added and sections were immersed in dH_2_O as brown staining appeared under light microscopy. Sections were counterstained with haematoxylin, dehydrated through graded ethanols and cleared with Xylene (Sigma) before coverslips were adhered using DPX. Staining intensity scores were determined by scorers blinded to the patient characteristics. A score of 0 denoted no positive brown COL4A1 staining and 3 was intense staining. Scores were averaged and plotted using Graphpad Prism 7. Images were captured using Olympus light microscope and CellSens software. Scale bars were added in Fiji Image software.

### Statistical analysis

All statistical analysis was performed in Graphpad Prism 7. All data is presented as mean ± standard error of the mean (SEM), unless stated otherwise. Normality of all data was determined by Shapiro-Wilk normality test to determine appropriate statistical tests.

Parametric data was analysed using two-tailed, paired or unpaired student’s t-tests. Non-parametric data was analysed using Wilcoxon signed rank test or Mann Whitney U-test.

Statistical tests performed include paired and unpaired Student’s t-tests for parametric data, or Wilcoxon signed rank tests and Mann Whitney test for non-parametric data. Alpha value was set at p < 0.05.

## Supplementary information

Supplentary information: miR-29c overexpression and COL4A1 downregulation in infertile human endometrium reduces endometrial epithelial cell adhesive capacity in vitro implying roles in receptivity.

## Data Availability

The data sets generated and/or analysed during the current study are available from the corresponding author on reasonable request.
