# Supplementary material for: miR-29c overexpression and COL4A1 downregulation in infertile human endometrium reduces endometrial epithelial cell adhesive capacity in vitro implying roles in receptivity
Source: Sci Rep. 2019 Jun 14;9:8644. doi: 10.1038/s41598-019-45155-6 (PMC6572831; doi:10.1038/s41598-019-45155-6)
Supplement: Supplementary file 1 — Supplentary information: miR-29c overexpression and COL4A1 downregulation in infertile human endometrium reduces endometrial epithelial cell adhesive capacity in vitro implying roles in receptivity. [file 41598_2019_45155_MOESM1_ESM.docx]

Supplementary data:

miR-29c overexpression and COL4A1 downregulation in infertile human endometrium reduces endometrial epithelial cell adhesive capacity in vitro implying roles in receptivity.

Meaghan Griffiths^1,2,3^, Michelle Van Sinderen^1,2^, Katarzyna Rainczuk^1,2^, and Evdokia Dimitriadis^1,2,4^*

^1^ Embryo Implantation laboratory, Hudson Institute of Medical Research, Clayton, Victoria 3168, Australia

^2^ Department of Molecular and Translational Medicine, Monash University, Clayton, Victoria 3800, Australia

^3^ Department of Anatomy and Developmental Biology, Monash University, Clayton, Victoria 3800, Australia

^4^ Department of Obstetrics and Gynaecology, University of Melbourne, The Royal Women’s Hospital, Parkville, Victoria 3010, Australia

*Corresponding author:

Evdokia Dimitriadis

University of Melbourne

The Royal Women’s Hospital, 20 Flemington Road, Parkville, Melbourne, Victoria 3010, Australia.

Phone: +61-3-83452215

Email: eva.dimitriadis@unimelb.edu.au

Supplementary Table T1. Patient characteristics for Early Secretory samples. (n=7/group).

|  | Fertile | Infertile | p-value |
| --- | --- | --- | --- |
| Age | 35.14 ± 3.73 | 36.14 ± 1.70 | 0.99 |
| BMI | 23.33 ± 1.13 | 23.93 ± 2.34 | 0.99 |

Supplementary Table T2. Patient characteristics for Mid-secretory samples. (n=6-8/group).

|  | Fertile | Infertile | p-value |
| --- | --- | --- | --- |
| Age | 36.50 ± 2.55 | 35.25 ± 1.99 | 0.99 |
| BMI | 27.65 ± 2.07 | 25.96 ± 2.72 | 0.95 |


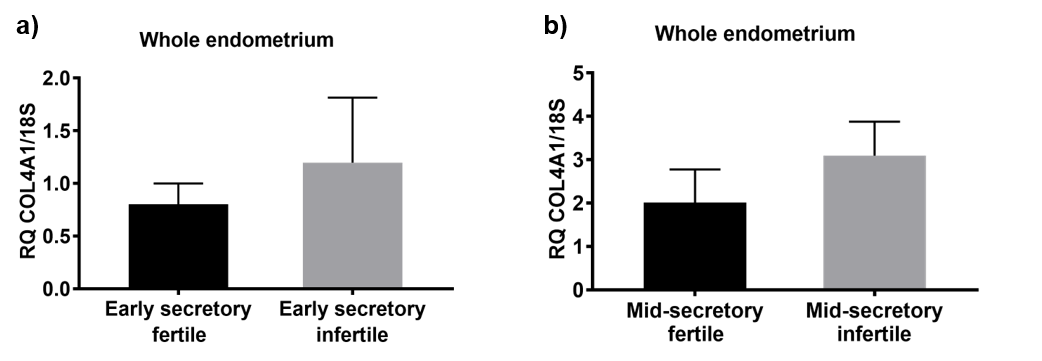


Supplementary Figure S1. A) COL4A1 mRNA levels in early secretory phase whole endometrium from fertile and infertile women. B) COL4A1 mRNA levels in mid-secretory phase whole endometrium from fertile and infertile women. Data are mean ± SEM, Shapiro-Wilk test for normality followed by Mann Whitney test for significance. (A. p>0.99, B. p=0.11). n=3-5/group.

Supplementary Figure S2. COL4A1 protein semi-quantification of the stromal and endothelial compartments in early (a-b) and mid-secretory (c-d) phase fertile and infertile endometrium. Data are presented as mean ± SEM. Sharpiro-Wilk normality test followed by Mann Whitney test for non-parametric data, or unpaired t-test for parametric data (a: p=0.28, b: p=0.93, c: p=0.78, d: p=0.61). n=7-8/group.


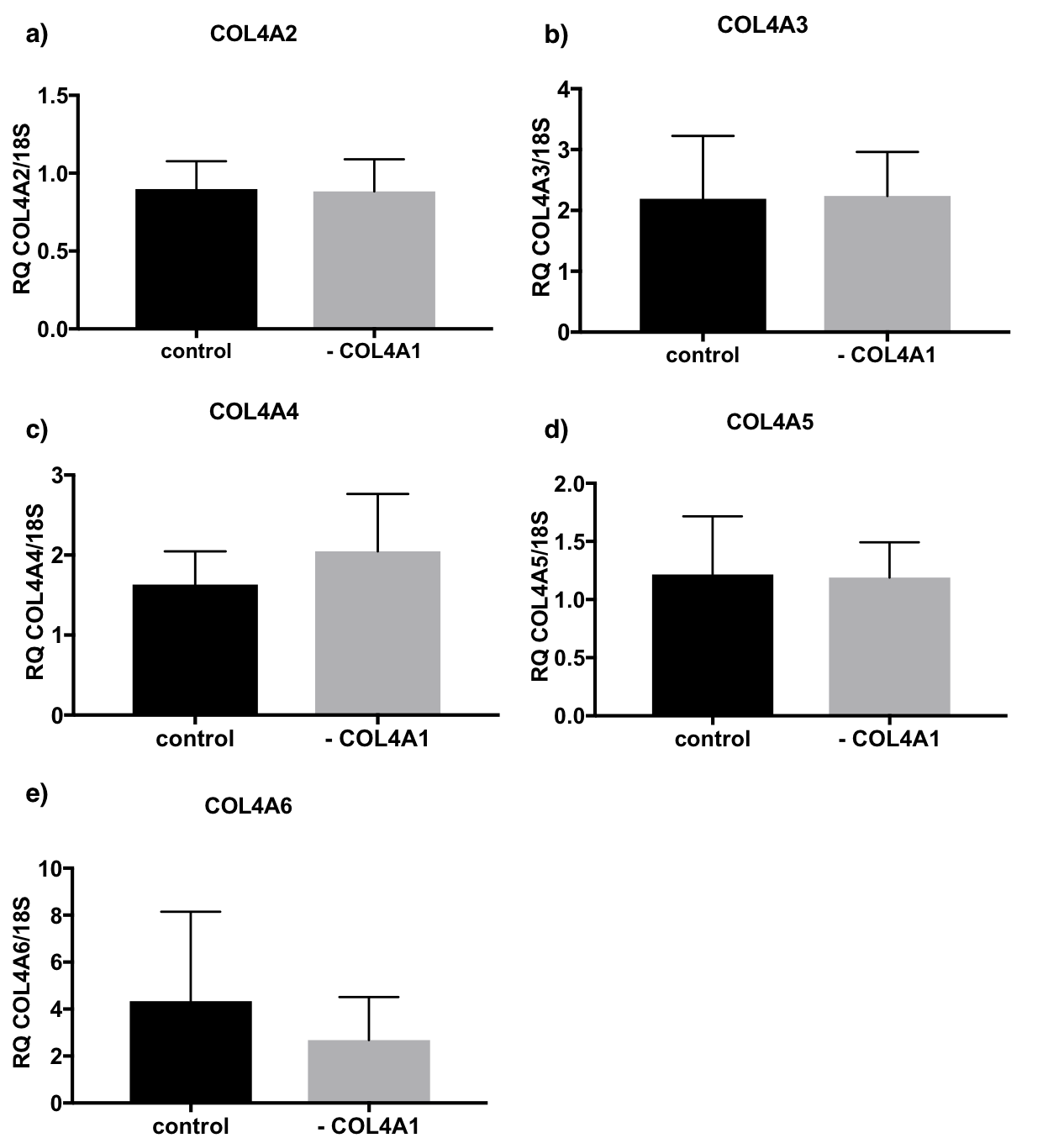


Supplementary Figure S3. Confirmation of COL4A1 siRNA specific knock down by assessing mRNA expression of Collagen type IV alpha chains 2-6 in HEEC transfected with COL4A1 siRNA. No change seen in expression of COL4A2-6 in HEEC where COL4A1 is reduced. Data are presented as mean ± SEM. Shapiro-Wilk normality test, followed by Wilcoxon signed rank test for non-parametric data, or paired t-test for parametric data (a: p=0.90, b: p=0.81, c: p=0.41, d: p=0.94, e: p=0.84). n=6 HEEC.
